# Lessons of kuru research: background to recent studies with some personal reflections

**DOI:** 10.1098/rstb.2008.0121

**Published:** 2008-11-27

**Authors:** John Collinge

**Affiliations:** MRC Prion Unit and Department of Neurodegenerative Disease, UCL Institute of Neurology, National Hospital for Neurology and NeurosurgeryQueen Square, London WC1N 3BG, UK

**Keywords:** kuru, prion, public health

## Abstract

The widespread exposure of the UK population to bovine spongiform encephalopathy prions, and the potential consequences for public health, led to a renewed interest in kuru, the principal example of epidemic human prion disease. Kuru research in Papua New Guinea was expanded to study the range of incubation periods possible in human prion infection, to investigate maternal and other possible natural routes of transmission, to characterize genetic susceptibility and resistance factors and to gain insights into the peripheral pathogenesis of orally acquired prion disease in humans. Although now essentially over, the kuru epidemic continues to provide important lessons.

It was in the early 1990s, when I was a junior doctor training in neurology and investigating genetic susceptibility to CJD and other prion diseases, that kuru became of increasing interest to me. The first mutations in the prion protein gene had been identified in 1988–1989 and the evidence suggested that these were causal for familial forms of spongiform encephalopathy and could be used in their definitive diagnosis (Owen *et al*. 1989; Collinge *et al*. 1989, 1990; Hsiao *et al*. 1989). This was somewhat controversial at the time but, if correct, offered a powerful direct route into understanding the pathogenesis of a neurodegenerative disorder. Furthermore, it had been known for some time that brain tissue from patients dying of these familial diseases could transmit the disease to laboratory animals by inoculation. This strongly suggested that some form of the so-called ‘protein-only’ hypothesis (Griffith 1967; Prusiner 1982) was likely to be correct, with major biological implications, not least owing to the existence of multiple ‘strains’ of the infectious agent known from long-standing work with sheep scrapie. Kuru was of course the first of the human prion diseases shown to be transmissible (Gajdusek *et al*. 1966), and of fundamental importance in the development of the field, but was largely thought to be of historical interest by this time.

However, against the background of these genetic advances and growing interest in the unique biology of these rare forms of human neurological disease was an emerging crisis that would unfold to worldwide prominence. The arrival of bovine spongiform encephalopathy (BSE; Wells *et al*. 1987) led a new impetus to understanding diseases long known to be transmissible, at least experimentally, between, as well as within, mammalian species. BSE rapidly developed into a major epidemic among UK cattle, estimated to have infected more than 2 million animals (Ghani *et al*. 2002), and was being increasingly recognized in other countries. The dominant view at that time was that BSE was simply scrapie in cattle. Cattle feed included meat and bone meal prepared from the rendered carcases of ruminants, which would have included scrapie-infected sheep. Since it was known that sheep scrapie had been around for centuries as a relatively common disease without apparent consequences for human health (epidemiological studies had shown no link between CJD and scrapie prevalence (Brown *et al*. 1987)), it was argued that BSE would also be no threat to human health. This view was increasingly challenged by the arrival and recognition of novel prion diseases in captive exotic ungulates and then, notably, in domestic cats *ca* 1990 (Wyatt *et al*. 1991). Cats had not hitherto been affected by these diseases. Indeed this was sufficiently high profile for the leading pet food manufacturer to rapidly withdraw an advertising campaign for their cat food bearing the slogan ‘what bowls a cat over?’ It became apparent that BSE, whether or not it had originated from some form of sheep scrapie, had a different host range and crossed between some species relatively easily by the oral route. Whether or not the host range of this novel infectious prion would involve humans could not be predicted but was clearly entirely possible. Furthermore, the population exposure to BSE prions was large, particularly before the ban on dietary use of specified offal (notably the brain and spinal cord) was introduced and effectively enforced. Bovine-derived materials had also been widely used in the manufacture of medicines, vaccines and many consumer products, including cosmetics. The key issue was the effectiveness of the so-called ‘species barrier’, known to limit cross-species transmission, which would determine the size of any corresponding human epidemic were BSE to be a human pathogen. Such barriers could not be predicted, and in laboratory animals are experimentally determined. Clearly, this was not possible in humans, and the effectiveness of the BSE-to-human barrier was therefore quite unknown. The molecular basis of such barriers was then beginning to be studied experimentally using transgenic mice (Prusiner *et al*. 1990) and we had set up long-term comparative transmission studies in ‘humanized’ transgenic mice to investigate the ability of BSE prions to propagate using human prion protein to address this key issue (Collinge *et al*. 1995).

Kuru, of course, provided the principal experience of an epidemic human prion disease, and one that had apparently been transmitted predominantly by the oral route. The prevailing assumption at the time was that kuru had effectively disappeared following a long decline in the epidemic after the interruption of its route of transmission: consumption of the deceased by their relatives at mortuary feasts. However, I had heard that, in fact, cases were still occurring, suggesting remarkable incubation periods. While the disease had been very extensively investigated in a series of seminal studies outlined elsewhere in this issue, it seemed that further study might yield specific information of relevance to these growing public health concerns.

With this evolving background, I wrote to Michael Alpers, then Director of the Papua New Guinea Institute of Medical Research (PNGIMR), in Goroka in 1992. Although I was by then very familiar with his published work on kuru, I had never met Michael. I explained my interest in kuru and asked whether indeed cases were still occurring and also whether it might be feasible to conduct an autopsy to collect tissues for the study using the newly emerging techniques. Michael responded by return of mail and said he had read my work, he would be happy to collaborate and that perhaps six to eight cases of kuru were still occurring annually. He suggested sending someone out to work in the field for six months initially. When I subsequently discussed what sort of person I should send, he replied ‘up to you, but do not send someone wearing a pith helmet’. ‘Oh, and you have to come too’. With this insightful advice, a long-term collaboration and friendship started.

Funding was of course then required. I was fortunate at that time to be funded as a Fellow of the Wellcome Trust (then directed by Bridget Ogilvie, who kindly spoke at the kuru symposium). The programme manager responsible for my research funding was David Gordon. The Wellcome Trust was to be enormously supportive to me personally as well as professionally during the subsequent BSE crisis. I went into the Trust offices on the Euston Road in London to see David to ask whether they might fund a small project on kuru. I discussed my rationale and outline plans and received an enthusiastic and characteristically straightforward reply. I was asked to draft a short application as a supplement to my fellowship to fund the project but was told ‘do not spend a lot of time on this, a couple of paragraphs will do, and just do some back of the envelope costings’. A few weeks later, the project was funded although at a level higher than that I had asked for; the Trust felt I had not asked for as much as I would need.

The next step proved more difficult and much more time consuming: finding the right person to send to Goroka. My first thoughts were to recruit a young clinician, preferably a pathologist. It became apparent that the rigidity of postgraduate medical training made this problematic. A distinguished pathologist colleague contacted me and suggested another approach, recruiting a nurse with extensive experience working in remote environments who could be trained to take on other responsibilities. My colleague had someone in mind and shortly thereafter introduced me to Jerome Whitfield. Jerome had an impressive, if unorthodox, curriculum vitae having worked as a paramedic in the army and with wide experience as a medical aid worker in developing countries. We talked for an hour or so in my office at St Mary's Hospital and it was apparent that Jerome was ideal and someone I felt would get on with everyone in what was undoubtedly going to be a challenging and wide-ranging role. He was also, as I was soon to learn to my cost in hopeless attempts to keep up with him on fieldwork, extremely physically fit, even, as I came to discover, by Fore standards. He agreed to go out to Papua New Guinea (PNG) for six months. Of course Michael knew well, and I soon learned, that the scientific objectives we had discussed would take far longer. Jerome would often say to visiting colleagues over a beer when we met in Goroka: ‘John told me I would be here for six months, and here I am a decade later’. Fortunately, the Wellcome Trust continued to generously support the project and Jerome was beginning to take to PNG.

In 1996 of course, the prion field exploded. During 1995, we had been involved in the investigation of three remarkably young patients (two teenagers and a woman in her 20s) who were neuropathologically diagnosed as CJD. Sporadic CJD was a disease of late adult life and exceedingly rare below the age of 40. There was no history to suggest iatrogenic exposure to human prions and we had sequenced the prion protein gene in all three and found no causal mutations (Bateman *et al*. 1995; Britton *et al*. 1995; Tabrizi *et al*. 1996). Each case generated massive media interest and made front page headlines in the UK. Print and broadcast journalists asked on each occasion ‘is this BSE?’ I answered, honestly, that I did not know and that you could not reach conclusions on individual cases but the occurrence of further similar cases would be very concerning. Following the third case, I was asked how many would have to be seen before it could be concluded this was BSE. I did not want to be drawn on that, but privately, and instinctively rather than on any formal statistical analysis, felt a fourth such extraordinary case in the UK following soon after would be enough at least to begin working on the assumption this was BSE-related with respect to public health considerations. We set up transmission studies into humanized transgenic mice of two of the cases in November 2005, so that these could be compared with our growing body of data on the transmission characteristics of the various forms of ‘classical’ CJD (Hill *et al*. 1997). Such experiments, which may not in any case have provided clear answers, were very time consuming and likely to take 2 years. The key issue would be to determine the prion strain type causing these cases and whether it was similar to that causing BSE in cattle.

In December 1995, I was invited by the Chief Medical Officer to join the UK Government's Spongiform Encephalopathy Advisory Committee (SEAC) and attended its next meeting in January 1996. At this meeting, I heard that two further extremely young cases had been identified by the National CJD Surveillance Unit, making a total of five. My own view then was that these cases were likely to be related to BSE exposure and we must work on this basis and immediately review measures in place to protect public health. This was not a view shared by all on the committee. By March, a total of 10 cases had been documented by the Surveillance Unit, all having a new pattern of neuropathology, which was presented to the committee by Professor Ironside, and SEAC concluded a new form of CJD had emerged in the UK probably related to BSE exposure, leading to the Government announcement in the House of Commons on March 20. This became known as ‘new variant’ CJD (Will *et al*. 1996). We considerably intensified ongoing work in my research group attempting to distinguish prion strains using rapid molecular methods and were able to distinguish the disease-associated prion protein (PrP^Sc^) type in new variant CJD from classical CJD types and showed it had a molecular signature closely similar to that causing BSE in cattle (Collinge *et al*. 1996). This experimental evidence that new variant CJD was indeed caused by a BSE-like strain was further supported by biological strain typing experiments in transgenic and conventional mice the following year (Bruce *et al*. 1997; Hill *et al*. 1997).

I was asked by the Medical Research Council (MRC) to expand my research group and develop an MRC Unit to work on prion disease, both to tackle the public health issues posed by vCJD and to study the fundamental molecular biology of prions and its wider implications. The MRC Prion Unit was formed in 1998 and led to a widening of our research on kuru, interest in which had redoubled since the arrival of new variant CJD. In particular, kuru provided a unique opportunity to study the range of incubation periods possible in human prion infection, investigate maternal and other possible natural routes of transmission, characterize genetic susceptibility and resistance factors and gain insights into the peripheral pathogenesis of orally acquired prion disease in humans.

The scientific objectives of our kuru research programme were as follows: to identify and study all remaining kuru patients and document the maximum incubation periods; to provide further data for accurate epidemiological modelling of the kuru epidemic to estimate key epidemic parameters; to document mortuary feast practices and traditional beliefs of the aetiology of kuru by interview of surviving participants and other members of the Fore community; to study the clinical features of current kuru patients and compare clinical and other diagnostic features with other human prion diseases, notably iatrogenic and variant CJD; to investigate any evidence of maternal or other routes of kuru transmission; to identify genetic susceptibility factors to kuru by study of recent patients and archived samples, long-term survivors of multiple feast exposures and the normal Fore and adjacent (exposed and unexposed) populations; to study the peripheral pathogenesis of kuru and tissue distribution of infectivity; and investigate the possibility of sub-clinical prion infection by analysis of autopsy tissues from patients and elderly exposed, but clinically unaffected, individuals.

Jerome led the re-establishment of research infrastructure and active kuru surveillance under Michael's guidance. A field base was established in Waisa in the South Fore and the PNGIMR house in the village refurbished, modernized and equipped to support the research programme (figure 1). The house, which had been rarely used, required considerable work to make it liveable and Jerome then established a garage, and fuel and equipment stores. Solar power was installed for lighting and the recharging of batteries. A small adjacent laboratory, for the fractionation and freezing of blood samples, was built and fully equipped with lighting, water supply, hand-powered centrifuge and a −40°C freezer. A team of local fieldworkers and kuru reporters was augmented, equipped and employed long term via the PNGIMR to assist with the project. A specially adapted Land Rover vehicle to support the project was shipped from the UK and a helicopter landing area was established by Jerome in the village for use of charter helicopters when road conditions were hazardous or impractical. The PNGIMR kindly provided an office base and administrative support for the project in Goroka.

The fieldwork has involved Jerome and his team walking thousands of miles in arduous terrain in poor weather conditions at high altitude in the Eastern Highlands Province (the Fore area is largely around 7000 feet above sea level; figure 2). Poor (to appalling and impassable in the wet season) road conditions hampered work (figure 3). The roads have significantly deteriorated over recent years. It takes 36 hours to cover 40 km in the wet season with heavy vehicle rescue equipment and a six-man team. Local politics often caused long delays to fieldwork. Local security issues intermittently affected our ability to travel to and from the field site. Under these field conditions, Jerome and his team documented all cases of kuru since 1996.

Working in PNG involves particular challenges and responsibilities with logistical, political and ethical issues to manage. Of course, the project was built entirely on the earlier work on kuru by Michael and the PNGIMR. Formal ethical approval was obtained from the PNG Medical Research Advisory Committee as well as from our ethics committees in London, but critically important from both the ethical and operational aspects was the full participation in the project of the communities involved. This involved both the Fore communities and neighbouring kuru-affected and unaffected linguistic groups from which we collected blood samples for comparative genetic studies. This was established and maintained through discussions with village leaders, communities, families and individuals (figure 4). The project adopted the usual procedures of research projects such as this one in PNG, where it is expected that in the course of studying human disease in the community some form of development, human, social or structural, will be achieved. We adopted the principles and standard practice of the PNGIMR in the conduct of the project in the field. It would be wrong to build up a strong participatory relationship with the communities and when the project was over leave nothing behind. Obviously, medical and nursing staff from the Unit also provided help, working where possible with local clinic facilities, to provide medical care. Kuru was thankfully by this time a rare cause of disease in these communities, who however faced major challenges with conventional infectious diseases (e.g. pneumococcal pneumonia, typhoid and malaria) and trauma, which we do our best to assist with.

We aimed to establish good relationships with local communities, based on mutual trust and respect of different beliefs as to the aetiology of kuru. Ongoing and regular discussions with the local community and its leaders are a vital part of the project. We have held medical clinics in remote areas and provided emergency medical treatment when required (figure 5). Seventy per cent of patients are paediatric cases who suffer from a wide range of infectious diseases. We have assisted the local Ivingoi Health Centre with medicine, diagnostics and casualty evacuation by car and helicopter. We have carried out complete treatment of malaria in villages affected by seasonal malaria outbreaks, and project personnel were trained for house spraying of insecticides in malaria-affected areas; spraying starts in the wet season with equipment and supervision provided by the PNG Provincial Malaria Unit.

We have assisted in the establishment of pre-schools in several villages and in the transport of building and school materials. The schools are run on a local basis with support from the Government and helped by funding from a UK-based charity and have proved to be sustainable (figures 6 and 7). With help from the British High Commission in Port Moresby, we established safe water supplies in a number of villages.

My first visit to the Fore was relatively brief, with only a few days in Waisa, but made a lasting impression on me. The terrain was staggeringly beautiful but made for challenging walking between villages. The warmth of the welcome, and generosity and openness of all I met was moving and inspiring. I presented a specially commissioned trophy to be used in the local rugby competition and was in turn presented with a formidable Fore bow with a range of arrows and instruction in their use. I have been fortunate in having been able to make annual trips since to see suspected patients when possible and to regularly attend the annual PNG Medical Symposium. Indeed these visits to the Fore and PNG more generally have become an important, highly educational and intensely rewarding part of my professional life. It also has led to ever-increasing respect and admiration for Michael Alpers (figure 8), with whom it has been nothing other than a privilege for me to collaborate. The respect and affection with which he is held by the Fore and more widely in PNG is profound, and appropriately so.

Soon after the events of 1996 in the UK, the BBC made two programmes on BSE and variant CJD as part of the science documentary series ‘Horizon’, in which the Unit featured heavily. We were able to use extracts from these programmes as teaching material and showed video at Ivingoi mission station where a generator and video player facilities existed. There was great interest in vCJD and the obvious similarities to kuru. Indeed some began to refer to vCJD as ‘English kuru’. On one occasion while Jerome and I attempted to explain the project to the community in a village in the South Fore, an elderly gentleman leaning quietly against a house interjected saying words to the effect of ‘You know, maybe kuru is not caused by sorcery after all if the white man gets it since we know they do not do sorcery’. A very thoughtful comment but with unfortunately a misplaced respect for the rationality of Europeans. I often found it worth asking those back in the UK, who express surprise that the Fore considered kuru to be caused by sorcery, whether they read horoscopes. Of course, magical or religious thinking about disease causation and cure permeates all human cultures, however medically advanced.

In another village, a man suggested a research project to me. He proposed using actual and mock sorcery bundles to test the sorcery theory of kuru and volunteered himself to be the subject. His outline protocol was not conceptually different from a placebo controlled trial. I found his proposal to use an experimental test very impressive, but, in addition to ethical considerations, did not relish putting this before the Neurosciences Board of the MRC.

Progress to date against our kuru research aims has been reported elsewhere (Mead *et al*. 2003; Collinge *et al*. 2006; Wadsworth *et al*. 2008*a*) and in this issue (Alpers 2008; Brandner *et al*. 2008; Collinge *et al*. 2008; Mead *et al*. 2008; Wadsworth *et al*. 2008*b*; Whitfield *et al*. 2008). Little of this work would have been possible without the generous collaboration of the Fore communities who played a key role in helping us understand a public health problem that was emerging in Europe but then disappearing from their community. Others from the UK assisted Jerome and his local team of kuru reporters for varying intervals, including Edward McKintosh, Eddy Lagan, Andrew Jackson, Toby Bentley, Ian Calder, Francesco Scaravilli and Adam Frosh. My distinguished neurological colleague from Queen Square, Dafydd Thomas, also assisted with assessment of patients. Many others were involved and are recognized by authorship and acknowledgement in the published papers. It is also vital to acknowledge the continual support we have received from the PNGIMR directors who have followed Michael: John Reeder and now Peter Siba.

One unexpected finding from our work on genetic susceptibility to kuru was the evidence for strong worldwide balancing selection acting at the prion protein gene locus during the evolution of modern humans and consistent with previous kuru-like epidemics in human prehistory, when cannibalism was thought to be widespread (Mead *et al*. 2003). It is also instructive to reflect, as has been pointed out previously, that use of tissue grafts and pituitary hormones derived from human cadavers, which have led to several hundred ‘iatrogenic’ cases of CJD in the USA, Europe and Japan (Brown *et al*. 2006), could be considered a modern western counterpart of cannibalism. Even with this wider perspective, the word cannibal remains highly pejorative and it would be preferable if an alternative term could be found to describe the ritual mortuary practices of the Fore.

One very special event to recollect here took place at the annual PNG Medical Symposium that was held in Goroka in 2005. While research communications on the project have been made regularly at these annual gatherings of the PMG medical profession, a special session was held on kuru at the 2005 meeting. Most importantly, this allowed several Fore members of the research team to present their work and their own personal experiences of kuru. The Fore presentations were made with clarity and great dignity and I think it is fair to say this had a profound impact at the meeting.

A further important recollection for me was of the visit of several Fore to the MRC Prion Unit and the National Prion Clinic (which was then based at St. Mary's Hospital in London). This had been arranged so that representatives on the Fore communities could see for themselves the research back in London and meet with the researchers. The visit had a major effect on my staff at the Unit and enduring friendships were forged. The Fore were keen to learn about vCJD and, importantly, it gave the opportunity for a meeting with someone who had lost a child to vCJD (figure 9). While I was aware of some striking clinical similarities between vCJD and kuru, it was fascinating as a clinician to hear discussions of the early features of both kuru and vCJD being discussed by relatives and the recognition during the discussions of further similarities of the two diseases in their prodrome and early clinical phases.

Kuru has taught us many lessons. The epidemic probably arose from consumption of an individual dying of sporadic CJD (a disease which appears to occur as a rare spontaneous disease in all human populations) by his or her kinship group, followed by successive rounds of recycling within the community as others developing or incubating the disease died and were themselves consumed at mortuary feasts (Alpers & Rail 1971). Recent strain typing of kuru prion isolates supports this view (Wadsworth *et al*. 2008*a*). Undoubtedly, the BSE epizootic also occurred owing to recycling of BSE prion-infected cattle into cattle feed. Again the initiating source of infection cannot be known with certainty, but there is little direct evidence to support a sheep scrapie origin and it may too have arisen from recycling of a sporadic prion infection of cattle. While some cases of sporadic CJD may relate to unidentified environmental exposure to human or animal prions, most are thought to relate to the rare spontaneous production of prions. There is no reason to suppose such a process, which has been well documented with yeast prions (Wickner *et al*. 2004), would be unique to humans, and sporadic prion disease probably occurs in all mammalian species, perhaps all vertebrates. Intraspecies recycling (cannibalism) of captive animals is therefore to be avoided as it risks an epidemic from amplification of naturally occurring prions. Use of such material to feed another species also carries some risk. While this will be mainly to closely related species, transmission barriers cannot yet be predicted for a new strain and may on occasion be surprising. BSE was of course surprising in its promiscuity and this may relate to selection of a particularly thermodynamically stable strain by the rendering (industrial cooking) process used to prepare meat and bone meal (Collinge & Clarke 2007).

Discussions are now taking place in the European Union on relaxation of the various control measures in place affecting animal feed. While some relaxation of especially precautionary measures may be appropriate, it is important that the lessons of kuru are not forgotten again. Policy makers, understandably under commercial pressures to relax controls, must be clearly advised about the potential consequences of recycling within the same or closely related species and it is important for all those who have learned the lessons of kuru to ensure this message is clearly expressed and not lost with the passage of time and turnover of policy makers. Although considerable uncertainties remain about the extent of pre- and sub-clinical infection of BSE-exposed human populations, the number of clinical cases has been thankfully relatively small (approx. 200). This suggests the presence of an effective transmission barrier to BSE prions in humans. Kuru reminds us, however, that human prion disease epidemics, even in the absence of such barriers, span over half a century (Collinge *et al*. 2006). The effect of transmission barriers is to extend both the mean and range of incubation periods considerably. Of course, secondary (human-to-human) transmission of vCJD prions by blood transfusion has now been documented and appears a worryingly efficient route (Wroe *et al*. 2006); many are also known to have been exposed to blood products and surgical instruments potentially contaminated with vCJD prions.

There is no known precedent for the widespread high-level exposure of a large human population to a novel animal prion strain as occurred with BSE. Further, it is entirely possible that a new prion zoonosis, should a return to cannibalistic recycling of animal tissues be allowed, might have a significantly less effective barrier to transmission to humans than does cattle BSE. The Fore have been extremely generous in allowing us to delve further into their tragedy and learn from their experience to better inform risks in Western societies. It is to be hoped that these lessons of kuru are now firmly learned by those responsible for public and animal health worldwide.

## Figures and Tables

**Figure 1:**
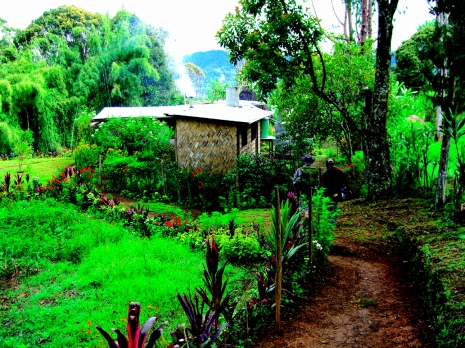
Field research house and laboratory in the village of Waisa, South Fore.

**Figure 2:**
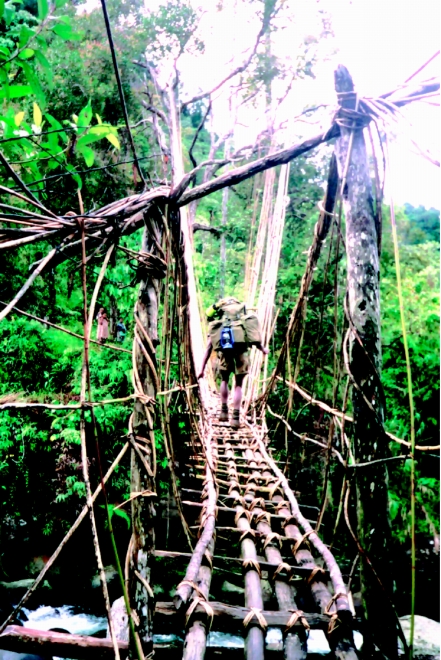
Jerome Whitfield on patrol.

**Figure 3:**
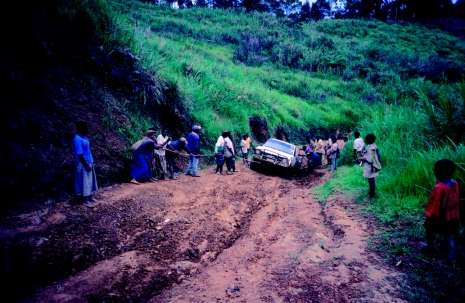
Typical recent conditions on Okapa road.

**Figure 4:**
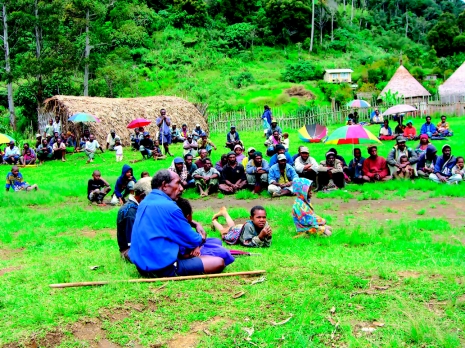
Village meeting in Waisa to discuss MRC/PNGIMR kuru research project.

**Figure 5:**
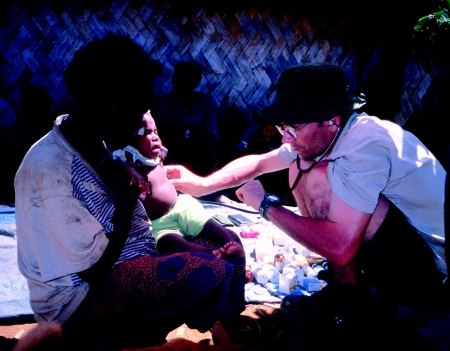
Jerome Whitfield at a local clinic.

**Figure 6:**
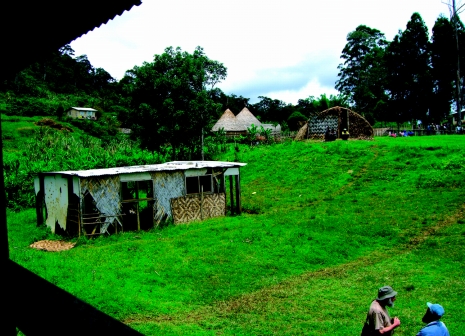
Old school building in Waisa.

**Figure 7:**
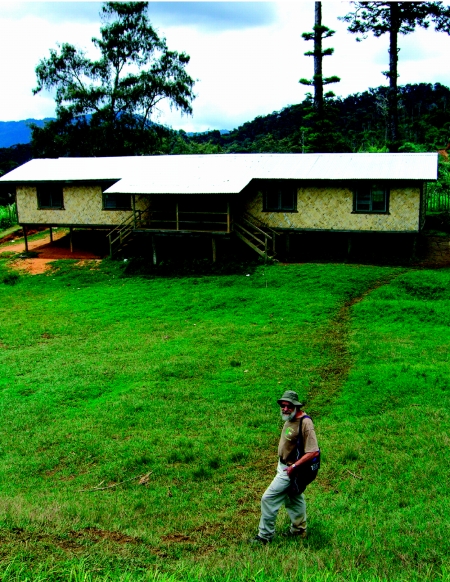
New pre-school building.

**Figure 8:**
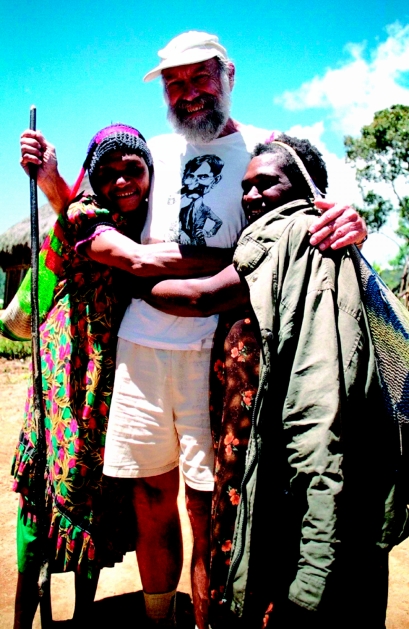
Michael Alpers and Fore greeting.

**Figure 9:**
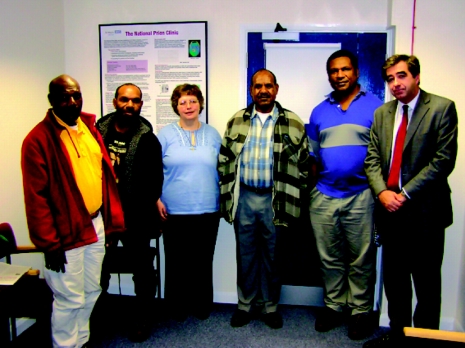
Visit of Fore representatives to National Prion Clinic and their meeting with Mrs Gibbs.
